# Why and How Political Science Can Contribute to Public Health? Proposals for Collaborative Research Avenues

**DOI:** 10.15171/ijhpm.2017.38

**Published:** 2017-04-05

**Authors:** France Gagnon, Pierre Bergeron, Carole Clavier, Patrick Fafard, Elisabeth Martin, Chantal Blouin

**Affiliations:** ^1^ School of Administrative Sciences, TÉLUQ, Université du Québec, Quebec City, QC, Canada.; ^2^ Department of Social and Preventive Medicine, Université Laval, Quebec City, QC, Canada.; ^3^ Department of Political Science, Université du Québec à Montréal, Montréal, QC, Canada.; ^4^ School of Public and International Affairs, University of Ottawa, Ottawa, ON, Canada.; ^5^ Faculty of Nursing, Université Laval, Quebec City, QC, Canada.

**Keywords:** Public Policy, Public Health, Research, Healthy Public Policies, Political Science

## Abstract

Written by a group of political science researchers, this commentary focuses on the contributions of political science to public health and proposes research avenues to increase those contributions. Despite progress, the links between researchers from these two fields develop only slowly. Divergences between the approach of political science to public policy and the expectations that public health can have about the role of political science, are often seen as an obstacle to collaboration between experts in these two areas. Thus, promising and practical research avenues are proposed along with strategies to strengthen and develop them. Considering the interdisciplinary and intersectoral nature of population health, it is important to create a critical mass of researchers interested in the health of populations and in healthy public policy that can thrive working at the junction of political science and public health.

## Introduction

In recent years, an increasing number of scientific papers,^1-7^ books,^8,9^ essays, commentaries,^10-13^ guides and tools^14-16^ have been exploring different facets of public health policy. The link between science and politics is openly debated in the context of the growing interest in evidence-based public health practice or even evidence-based policy-making.^10,17,18^ The development of strategies to support the adoption of health-promoting public policies^14,15^ appears to many experts as essential for the future of public health. Political science approaches have also been used, more or less directly, to analyse public health problems related to the social determinants of health and health inequalities,^19-22^ as well as the promotion of healthy lifestyles (tobacco use, alcohol, obesity).^23-26^ Despite progress, close linkages between researchers in public health and in political science are a long time coming.

Public health researchers often criticize political scientists for being too “theoretical” in their approach. Political scientists, on the other hand, often consider public health researchers as having a “naive” understanding of political reality. Yet these generalizations do not justify the lack of rapprochement. In a context where the value of inter-disciplinarity and inter-sectoriality is increasingly recognised, there is a considerable room for improvement. The objective of this commentary is to outline why, and in what ways, political science can contribute to public health both as a field of inquiry, policy and practice.

What follows builds upon existing research as well as upon our own experiences in Canadian research, teaching, and knowledge-transfer institutions. Individually and collectively we have explored the intersection of public health and political science by means of congresses,^27,28^ workshop,^29^ deliberative forum,^30^ the development of a network of public policy researchers interested in population health; and, collaborative research reports.^31,32^ We now wish to advance our collective understanding and underscore the importance of further developing collaborations between researchers in the field of public health and political science. Our goal is to shed more light on the complex public health problems that confront public health officials, political leaders, and indeed the population at large.

To do this, we will first examine the main characteristics of public health and political science in order to highlight how they diverge regarding their interpretation of public policy, but also how they converge in terms of their common interest in understanding how healthy public policies are put into place. This examination will underline underexplored issues as well as the political variables that are rarely examined in research about healthy public policies. We will then propose avenues for research that deserve more attention if we are to advance our collective understanding of public health.

## Distinct Fields but Common Interests

Political science is usually associated with the: “study of the state, government and politics,”^33^ or with the analysis of power relations and of the relations between the rulers and those subjected to their rule (the nature of power, its foundation, how it is deployed, its objectives and effects). To put it another way, political science investigates: (1) politics – formal and informal political action, including political parties, interest groups and social movements; (2) policies – public policies which are the translation of collective choices and structure the allocation of resources; as well as, (3) the polity: formal political institutions and political systems.^34^ It includes different research domains such as comparative political systems, public administration, international relations, and policy analysis.

Public health can be defined as cross-roads of disciplines where medicine, epidemiology, and nursing converge and open up to social sciences such as psychology, anthropology, and sociology. It is also a professional and institutional activity aiming to protect and promote the health of the population thanks to interventions in different areas such as infectious diseases, environmental health or the promotion of healthy lifestyles. It is a field which relies on various bodies of knowledge for public action; its interventions can take many forms such as direct services to individuals or communities, diseases surveillance, or the development of health-promoting public policies.^35^ Despite early recognition of the political dimension of health and of the influence of the social and political environment of health,^36,37^ detailed study of interactions between health and politics are often neglected.^38^

In all countries, protection of the population from health risks is the traditional and main mission of public health. Health promotion has developed more recently, to various degrees and forms.^39^ It has close ties to the social sciences, including political science.^4,40^ This is particularly the case since the publication of the Ottawa Charter in 1986 which advocates for the adoption of health-promoting public policies^41^ and, more recently, with the advent of a “health in all” policies approach.^42^ However, there are divergences between the approach of political science to public policy and the expectations that public health often has about politics and policy-making. These divergent expectations are often an obstacle for collaboration between political scientists and public health experts.^7,43-45^ In summary, these divergent views include:

- The contrast between political scientists who seek to understand the policymaking process which leads to the adoption and implementation of public policies vs. public health specialists who seek to identify the steps to take in order to successfully advocate for health-promoting public policies. The former conceive of health and the complex challenges related to this domain as an area of study while the latter often perceive political science as a resource or an instrument to achieve healthy public policy.
- The recognition of the uncertainties and limitations of expert knowledge on public policies that is common in political science vs. the instrumental use of knowledge and expertise as a lever to promote health-promoting public policies. Research in political science has clearly shown that the making of public policy is neither a linear nor a uniquely reasoned process.^46-48^ Empirical work centred on public health issues has documented that the availability of scientific evidence alone is not sufficient to convince decision-makers of the relevance of a solution.^31,49-50^ Nevertheless, in the public health literature there continues to be calls for public health policy to be evidence-based or at least evidence-informed with little reference to the complexities of public policy-making.^51^
- The recognition of the plurality of interests in our societies^10^ vs. the public health assumption that health considerations are (or should be) the predominant determinants of public policy decisions.^52^ In the political science literature it is axiomatic that, when deciding about policy, governments must balance and reconcile multiple overarching goals and objectives that in addition to health issues, decision-makers also have to weigh economic and environmental challenges to name but two.
- The political science interest in analyzing the roles and responsibilities of various actors (including public health) within public institutions and state governance more broadly vs. a public health interest in using the roles and responsibilities of these public health actors to advocate for social and policy change. A political science perspective focuses on the integration of the public health function into the modern machinery of government which, when combined with overarching rules about public service neutrality and limited visibility, has the effect of constraining the policy advocacy by public health officials.^53^

Political science and public health thus often have different points of view about the process by which public policy is, or should be, formulated. But there is, nevertheless, common ground in a number of areas and both fields have a shared interest in actions that promote the public good. The public and institutional character of public health creates interest in institutions, public administration, and governance. The goal of health-promoting public policies creates an interest in policy analysis, ie, the analysis of the choices to be made, of the resources to be allocated, the knowledge to be used, and of the dynamics of actors trying to influence the content of public policies. Given these areas of common interest, it is important to identify the potential contributions of political science to a better understanding of some of the key challenges faced by public health.

## Potential Research Avenues

In order to conduct fruitful and innovative research, the expertise and knowledge developed by both fields is critical. With a view to providing a stronger foundation for our analysis of the complex issues confronted by public health drawing on the expertise and insights of political science, and also on our own collaborative research with public health experts, we believe that the following research avenues are both promising and practical:

- Sharing, application and exchange on the various conceptual models and schemes of interpretation related to the formulation, adoption and implementation of public policies in the *political context* of our societies. In thinking about public health policies there is a need to take into consideration the diversity of political variables which influence collective choices and the ethical dilemmas they raise, for example between individual freedom and the common good.^54^ To do this, the mechanisms of participatory or deliberative democracy can be used to supplement the existing institutions of representative government.^55,56^
- Analysis and discussion of the *functions of public health* with greater attention to the role of the state, of public administration, and of governance, at the local, national and global levels. What is also required is greater consideration of the dynamics between the local, national and global level. We also need to ask about the functions that public health actors plays at each level and the basis for their legitimacy.^57^
- More emphasis on *evaluation*, prospective and retrospective, of public policies that can allow, when needed, for adjustment in the health-promoting public policy. Program evaluation has already received some attention but public policy evaluation is less frequent, even though it is crucial to critically assess the choices made, the resources invested and their impact. This being said, the growing interest in health impact assessment (HIA) has led to some experiments and institutionalisation, particularly in Anglo-Saxon countries.^58-61^
- Greater recourse to the *comparative analysis* of regions, provinces, or countries to highlight the strengths and the limitations of the healthy public policy being considered. It is under-explored, but comparative analysis can be a fruitful source for policy learning across jurisdictions and organisations.^62,63^

It is possible to press ahead and use diverse strategies to strengthen and develop those research avenues. Those strategies include holding exchange workshops involving different generations of researchers and practitioners; joint interdisciplinary and intersectoral research workshops on public policy case studies; and taking full advantage of existing experimentation and practices.

## Conclusion

In this commentary, we have underscored how it is critical to anchor the analysis of public health policies in the political science approaches and models. Clearly, tools and analysis have already been developed to better support the analysis of public policies. However, gray areas remain as to the most appropriate way of analyzing the political variables that sometimes interfere with making public health solutions a priority in the political system. We have targeted research avenues that would contribute to better understand the dynamics among actors involved in collective decision-making related to health, at the intersection where knowledge and values often clash.

## Acknowledgements

The authors thank the Quebec Population Health Research Network for its support to the Public Policy and Population Health Strategic Group. Thank you to the anonymous reviewers for their helpful comments on an earlier version of this article. All authors are members of the Public Policy and Population Health Strategic Group of the Quebec Population Health Research Network (QPHRN/RRSPQ), funded by the Fonds de recherche du Quebec_Santé (FRQ_S).

## Ethical issues

Not applicable.

## Competing interests

Authors declare that they have no competing interests.

## Authors’ contributions

FG, PB, CC, PF, and EM conceptualized the paper. FG wrote the manuscript, and all authors contributed to revision.

## Authors’ affiliations

^1^School of Administrative Sciences, TÉLUQ, Université du Québec, Quebec City, QC, Canada. ^2^Department of Social and Preventive Medicine, Université Laval, Quebec City, QC, Canada. ^3^Department of Political Science, Université du Québec à Montréal, Montréal, QC, Canada. ^4^School of Public and International Affairs, University of Ottawa, Ottawa, ON, Canada. ^5^Faculty of Nursing, Université Laval, Quebec City, QC, Canada.
